# Xanthogranulomatous osteomyelitis of ulna mimicking neoplasm

**DOI:** 10.1186/1477-7819-5-46

**Published:** 2007-04-30

**Authors:** Mahesha Vankalakunti, Uma N Saikia, Manoj Mathew, Mandeep Kang

**Affiliations:** 1 Department of Histopathology, Postgraduate Institute of Medical Education and Research, Chandigarh, India; 2 Department of Orthopedic surgery, Postgraduate Institute of Medical Education and Research, Chandigarh, India; 3 Department of Radiodiagnosis Postgraduate Institute of Medical Education and Research, Chandigarh, India

## Abstract

**Background:**

Xanthogranulomatous osteomyelitis often presents as a severe chronic inflammation associated with pain, fever, and leukocytosis. It may mimic carcinoma in the involved organs.

**Case presentation:**

A 50-year-old post-menopausal woman presented with a 2 year history of increasing swelling in the extensor aspect of her right forearm. Plain X-ray revealed an ill-defined expansile osteolytic lesion in the diaphysis of ulna. The gross, microscopic and ultrastructure findings of the curettage specimen was consistent with xanthogranulomatous osteomyelitis.

**Conclusion:**

This case highlights the rare occurrence of xanthogranulomatous osteomyelitis involving ulna, which can mimic as a primary or secondary bone tumors. A correct diagnosis can only be made on histopathological examination.

## Background

Xanthogranulomatous osteomyelitis (XO) is a specific type of chronic inflammatory process characterized by collection of foamy macrophages admixed with mononuclear cells [1]. On gross examination, this phenomenon can mimic carcinoma in the involved organs [2]. XO has characteristic findings, i.e., the presence of granular, eosinophilic, PAS positive histiocytes in the initial stages; the mixture of foamy macrophages and activated plasma cells; the presence of suppurative foci and hemorrhage. It has been encountered in various tissues viz. the gallbladder, kidney, urinary bladder, fallopian tube, ovary, vagina, prostate, testis, epididymis, colon and appendix. Very rarely, it can affect lungs, brain, or bone. Only two cases of xanthogranulomatous osteomyelitis have been described previously in the literature [3].

## Case presentation

A 50-year-old post-menopausal woman presented to the out-patient department with a bony hard swelling on the extensor aspect of her right forearm. The swelling was gradually increasing in size, over the past 2-years. She had sustained a fracture of the ulna 11-years ago treated by 4 weeks splintage.

**Figure 1:**
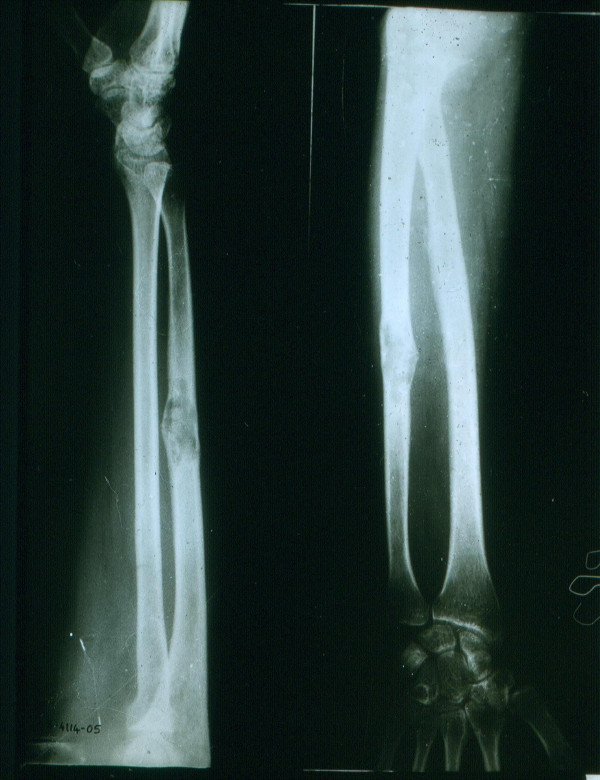
X-ray of postero-anterior and lateral view of right forearm showing an expansile lytic destructive lesion involving mid one-third of diaphysis of ulna, with ill-defined zone of transition.

On local examination there was a small non-tender prominence of 2 × 3 cm located in the middle one-third of the right ulna. Both anteroposterior and lateral radiographs revealed an expansile lytic destructive lesion involving the mid one-third of diaphysis of the right ulna, with an ill-defined zone of transition (Figure 1). No matrix calcification or periosteal reaction is seen. Surrounding soft tissues were normal. The patient had already been undergoing treatment for primary hypothyroidism, and benign positional vertigo. Other systemic examination was normal.

Serological examination revealed an elevated alkaline phosphatase of 338 IU/L (normal: 100–280 IU/L), other serum biochemistry including liver and renal function tests were normal. A general radiographic skeletal survey ruled out the presence of similar lesions at other sites. Serum parathormone estimation was within normal range and an ultrasound scan of the neck failed to reveal any thyroid or parathyroid abnormality. A clinical suspicion of primary bone tumor was entertained. The involved bone segment was curetted out and cancellous iliac crest graft interposed. A bridge-plating was performed and the forearm splinted for two weeks after which she was started on physiotherapy.

**Figure 2:**
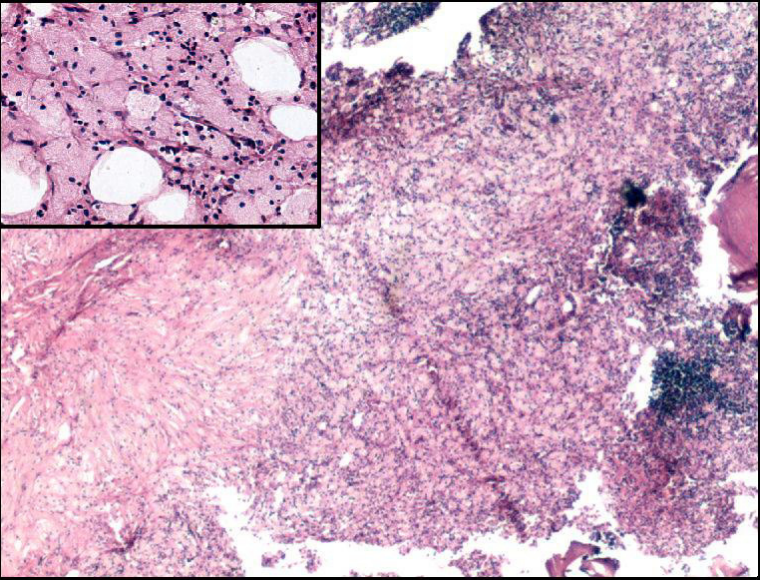
Light microscopy showing diffuse collection of foamy macrophages with nodular aggregates of lymphocytes (H&E, × 25). *Inset: *Higher magnification shows foamy macrophages having distinct cytoplasmic border, abundant pale granular cytoplasm and small pyknotic nucleus (H&E, × 240).

On gross examination the tissue was soft in consistency, yellowish in color with few bony particles. Histologically the lesion showed a diffuse infiltrate, composed of numerous foamy histiocytes admixed with inflammatory infiltrate (Figure 2). Focally nodular aggregates of mature lymphocytes were noted along with presence of plasma cells and neutrophils. Transversing capillaries and occasional multinucleated giant cells were also seen. No pigment was seen in the cytoplasm of macrophages. The tissue cultures failed to reveal any organisms. Ultrastructure examination confirmed the cells to be of fibrohistiocytic in origin. No Birbeck granules were demonstrated, thereby ruling out Langerhans cell histiocytosis. The postoperative period was uneventful and the patient was lost in the follow-up.

## Discussion

Xanthogranulomatous osteomyelitis (XO) is encountered in various tissues namely the gallbladder, kidney, urinary bladder, fallopian tube, ovary, vagina, testis, epididymis, colon and appendix [4-6]. Very rarely, it can affect lungs, brain, prostate, or bone. On gross and radiological examination, XO is a great mimicker of malignancy, forming mass lesions in gallbladder, kidney and prostate [7]. Histology of such lesions reveals its chronic inflammatory nature. The two previously described cases of XO by Cozzutto, were in patients in the first 2 decades of life and involved the first rib and the tibial epiphysis respectively [3].

With respect to the origin of foam cells, it was considered that the vast majority of foam cells were derived from monocytes/macrophages because they were invariably positive for KP1, HAM56, CD11b and CD68. It is a granulomatous disorder characterized by accumulations of macrophage foam cells and T cells. Delayed type hypersensitivity reaction of cell-mediated immunity may be implicated in the pathogenesis of XO [8].

A relationship between trauma and XO developing at the same site is conjectural.

The differential diagnoses include chronic recurrent multifocal osteomyelitis (CRMO), xanthoma, infiltrative storage disorder, malakoplakia, Langerhans-cell histiocytosis, fibrohisticotytic tumor, erdheim-chester disease and metastatic renal cell carcinoma [9-12].

We think that the biopsy should be performed in the management, apart from magnetic resonance imaging and computed tomography scan. However, the biopsy in the index case was not done due to technical reasons.

## Conclusion

This case is primarily presented due to its rarity. Xanthogranulomatous osteomyelitis has not been reported in the literature to the best of our knowledge. The importance lies in the differential diagnoses of XO which simulates a tumor-either primary or secondary, both on radiology and gross examination. A histopathological examination from the representative areas is needed to establish the correct diagnosis and hence imparting appropriate treatment.

## Competing interests

The author(s) declare that they have no competing interests.

## Authors' contributions

**VM, UNS**: Histopathological examination of submitted tissue and preparation of manuscript/photomicrographs; **MM**: Orthopedic surgeon; **MK**: radiological assessment of the case.
